# Flooding and Mental Health: A Systematic Mapping Review

**DOI:** 10.1371/journal.pone.0119929

**Published:** 2015-04-10

**Authors:** Ana Fernandez, John Black, Mairwen Jones, Leigh Wilson, Luis Salvador-Carulla, Thomas Astell-Burt, Deborah Black

**Affiliations:** 1 Faculty of Health Sciences, Centre for Disability Research and Policy, Brain and Mind Research Institute, The University of Sydney, Sydney, Australia; 2 Faculty of Health Sciences, Ageing Work and Health Unit, The University of Sydney, Sydney, Australia; 3 Faculty of Health Sciences, Discipline of Behavioral and Social Sciences in Health, The University of Sydney, Sydney, Australia; 4 School of Science and Health, University of Western Sydney, Sydney, Australia; University of Washington, UNITED STATES

## Abstract

**Background:**

Floods are the most common type of global natural disaster. Floods have a negative impact on mental health. Comprehensive evaluation and review of the literature are lacking.

**Objective:**

To systematically map and review available scientific evidence on mental health impacts of floods caused by extended periods of heavy rain in river catchments.

**Methods:**

We performed a systematic mapping review of published scientific literature in five languages for mixed studies on floods and mental health. PUBMED and Web of Science were searched to identify all relevant articles from 1994 to May 2014 (no restrictions).

**Results:**

The electronic search strategy identified 1331 potentially relevant papers. Finally, 83 papers met the inclusion criteria. Four broad areas are identified: i) the main mental health disorders—post-traumatic stress disorder, depression and anxiety; ii] the factors associated with mental health among those affected by floods; iii) the narratives associated with flooding, which focuses on the long-term impacts of flooding on mental health as a consequence of the secondary stressors; and iv) the management actions identified. The quantitative and qualitative studies have consistent findings. However, very few studies have used mixed methods to quantify the size of the mental health burden as well as exploration of in-depth narratives. Methodological limitations include control of potential confounders and short-term follow up.

**Limitations:**

Floods following extreme events were excluded from our review.

**Conclusions:**

Although the level of exposure to floods has been systematically associated with mental health problems, the paucity of longitudinal studies and lack of confounding controls precludes strong conclusions.

**Implications:**

We recommend that future research in this area include mixed-method studies that are purposefully designed, using more rigorous methods. Studies should also focus on vulnerable groups and include analyses of policy and practical responses.

## Introduction

Floods are the most common type of global natural disaster responsible for almost 53,000 deaths in the last decade [1, 2]. In Australia, for example, floods are the most expensive natural hazard experienced, leading to an average annual damage bill of over AUD $300 million [3]. In March 2013, the Climate Change Institute at the Australian National University released a report highlighting the increasing likelihood of flood events [4]. As such, the magnitude of the problem could increase in the future. The impact of floods on those who experience them can be significant. In addition to economic loss, detrimental short-, medium- and long-term effects on wellbeing, relationships and physical and mental health are common[1, 5]. Due to the long duration of mental health problems and their impact on other chronic conditions, it has been calculated that mental health problems are responsible for more than 80% of all the estimated Disability Adjusted Life Years (DALYs) attributable to floods in the United Kingdom between 2003 and 2008 [6].

A recent literature review examining the effects of flooding on mental health [5] highlighted a number of limitations of research conducted to date. These included the cross-sectional nature of the research and the lack of information on long-term effects of flood exposure. In addition, there was minimal systematic evaluation of flood mitigation and its impact on potentially promoting social capital in flood-affected communities. However, the review by Stanke et al [5] also had some limitations: it was not conducted systematically; was limited to papers written in English; and undertaken between 2004 and 2010; and included only quantitative papers, thus excluding information from qualitative studies. Qualitative data are crucial to a comprehensive understanding of the impact that floods have on the mental health of people affected and can be helpful in the design of policies and strategies to mitigate the impact of floods on mental health. Due to the complexity of health and social services planning, provision and delivery, it has been suggested that mixed-study reviews are critical to better planning of public health interventions [7]. To the best of our knowledge, there is a lack of reviews using mixed-method studies on this topic.

The review conducted by Lowe, Ebi and Forsberg on the impact of floods on health includes different types of flooding events such as floods caused by cyclones, typhoons or hurricanes, coastal inundations from storm surges, or tsunamis, which have different degrees of severity, frequency and impacts [8]. Of the 286 studies in this review only 38 were reviews of fluvial flooding – the most common cause of flooding. Consequently, relatively little is known about the specific impact on mental health of fluvial flooding, the most common type of flooding, caused by heavy rain in river catchments [8].

This paper addresses this gap by focusing on the mental health impact of floods caused by extended periods of heavy rain in river catchments. A systematic mapping review of the published literature in the last 20 years was conducted. This review follows the recommendations made both by the PRISMA group [9] and by the Evidence for Policy and Practice Information Group [10]. In addition, we aim to identify gaps in the research and develop recommendations that will aid in improving the research on the impact of flood in the mental health of affected communities.

## Methods

### Literature Search

We performed a systematic mapping review of the published literature on floods and mental health. Systematic mapping aims “*to describe the extent of the research in a field and to identify gaps in the research base*. *It identifies gaps in the research*, *where further primary research is needed*, *and areas where no systematic reviews have been conducted and there is scope for future review work”* [10]. Systematic mapping provides descriptive information about the ‘state of the art’ of a topic and a summary of the research conducted [10].

We carried out literature searches for this topic from January 1994 to May 2014, through PubMed, and Web of Science. The search was restricted to the past 20 years because from the early 1990s there has been an increasing focus on empirical evidence-based research and there was also a change in the description of mental disorders in the 4^th^ version of the Diagnostic and Statistical Manual of Mental Disorders (1994]. We used free text and words were restricted to title and abstract. The word “flood” has an asterix (*) added as a wildcard in to pick up plurals and other words such as “flooding” using Boolean logic. The search strategy was:

(depression (Title/Abstract) OR anxiety (Title/Abstract) OR posttraumatic (Title/Abstract) OR distress(Title/Abstract) OR psychological (Title/Abstract) OR mental (Title/Abstract)) AND (flood*)

Titles and/or abstracts of all citations were obtained for study selection. Citation indices and reference lists of retrieved articles were checked for additional studies not identified in the original database search.

### Study Selection

Studies were screened for inclusion in two phases. In phase 1, the first author reviewed the title and the published abstracts and selected a preliminary list of papers. Papers published before 1994 were excluded for two reasons: i) to have the most recent literature; and ii) to avoid possible differences in the description of mental disorders, as it was in 1994 that the 4^th^ version of the *Diagnostic and Statistical Manual of Mental Disorders* was published. All papers published from 1994 onwards were reviewed by at least two of the authors, who independently selected papers that they believed met the inclusion criteria for full-text reading. Where disagreement arose, the researchers discussed their different assessments and reached a consensus. In phase 2, the first author reviewed the full articles that met the eligibility criteria.

### Eligibility criteria

#### Population

All papers that focused on the effects of floods on mental health or the responses to ameliorate the effects of flooding on human mental health were included. Papers focusing on the impact of floods on the ecosystem were excluded.

#### Topic of the paper

We included all papers dealing with: (i) flood exposure; (ii) narratives related to floods and mental health; and (iii) interventions to ameliorate the impact of flooding on mental health. Papers were excluded that examined floods caused by hurricanes, typhoons, tsunamis and their resultant coastal inundations. Furthermore, papers examining the effects of non-flood natural disasters on mental health were excluded.

#### Outcome

All papers that considered any mental health measure as primary outcome of the impact of flooding were included. Therefore, papers in which mental health was not the final outcome but a covariate were excluded.

#### Study design

The systematic mapping included the following study designs: reviews; case-control studies; longitudinal studies; cross-sectional surveys; qualitative studies; risk modelling studies; and case studies. Clinical cases, letters, abstracts to congress, editorials and expert commentaries were excluded.

#### Language

We included all the papers written in English, Spanish, French, Italian or Portuguese.

### Data extraction

The first author extracted key features of the characteristics, methods, and outcomes of articles that met the inclusion criteria. Key features included the period of study and type of flood studied. For example specific episodes of flooding such as the January 2011 floods in Queensland or the 1993 floods in Missouri presented the cumulative effect of a number of different floods. The study design, demographics and a short summary of the results were reviewed by the co-authors.

### Risk of bias/ quality of the studies

The main aim of a systematic mapping review is to describe the state of the art of a topic. Risk of bias is done in a generic way by classifying the papers by type of study. In this systematic mapping review, the information derived from quantitative studies was discussed in terms of the overall bias associated with the different designs [10] and with respect to the main research focus. The qualitative studies were reviewed in terms of the research coherence and of the utility of the findings [11].

## Results

### Literature search and study selection

**Fig 1:**
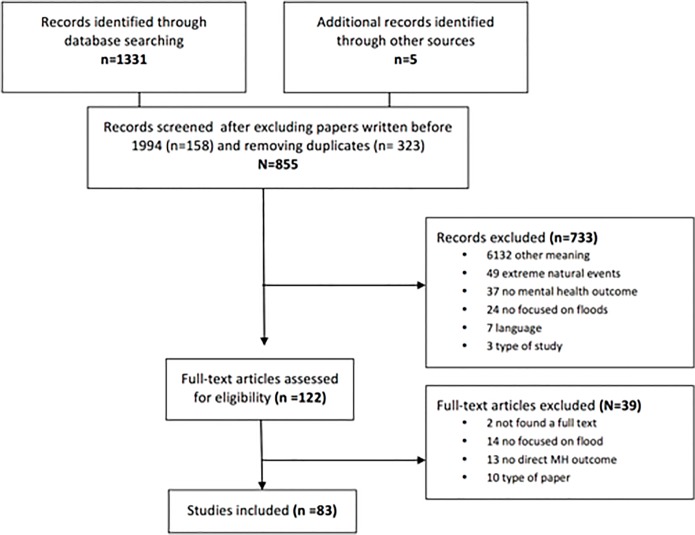
Flow Diagram.

The electronic search strategy identified 1331 potential papers. Five additional papers were identified through other sources. After removing duplicate studies, or those that were published before 1 January 1994, 855 were reviewed by reading the title or abstract. A total of 733 were excluded; 613 (84%) because they either had a non-mental health meaning of the word “depression” (e.g. meteorological or geological] or had the word “flood” with a different meaning (e.g. a type of psychological therapy called flooding). Amongst the 122 full-text articles assessed for eligibility, 83 were included for further study (Fig. 1). A summary of the papers selected can be viewed in the S1 Supporting Information.

### Characteristics of the studies selected

Table 1 provides a summary of the characteristics of the included manuscripts. Fifty-four of the manuscripts (65%) used quantitative methods. Of these, 29 were cross-sectional surveys without control groups (54%). Only 12 of the 54 (23%) studies included a control group and only eight studies (15%) utilised a baseline measure. Ten (12%) were qualitative studies dealing with the subjective experiences of flood survivors. Eleven (13%) reviews dealing with the effects of flooding on mental health were also included. Only two reviews were specifically focused on the impact of floods on mental health. However, these also included all types of flood-related events (tsunamis, hurricanes, typhoons). Finally, eight (10%) papers involved case studies, which mainly examined responses to a specific flood event, including relevant authorities responding to ameliorate the impact on mental health.

**Table 1:** Summary of the characteristics of included references

**Type of Study (references]**	%	N
Quantitative methods	**65%**	54
• Longitudinal study with baseline and control group [14, 47] (4% of quantitative studies)		2
• Longitudinal study (with baseline measure, without control group][43–45, 48, 58, 65] (11% of quantitative studies)		6
• Case-control study (without baseline measure)[13, 15–17, 42, 46, 50–53] (19% of quantitative studies)		10
• Cross-sectional survey (no baseline or retrospectively assessed, no control group) [18–41, 49, 54–57] (54% of quantitative studies)		29
• Longitudinal study without baseline measure and without control (monitoring of the impac) [12, 59–64] (13% of quantitative studies)		7
Qualitative methods[66–75]	**12%**	10
Case-Study[76–78, 80, 82–85]	**10%**	8
Reviews[1, 5, 8, 79, 81, 86–91]	**13%**	11
**Characteristics of the Sample**		
**Age**		
Child and Adolescent[21, 29, 36, 40, 45, 69]	7%	6
Adults (without distinction][12, 13, 15–19, 23–26, 28, 31–35, 37–39, 42, 43, 46, 49, 52, 53, 55–68, 70, 72–75]	54%	45
Older Adults[14, 44, 71, 87]	5%	4
All ages[1, 5, 8, 20, 22, 27, 30, 41, 47, 48, 50, 51, 54, 76–86, 88–91]	34%	28
**Characteristic of the floods**		
Specific[12, 13, 16–23, 27–46, 48, 49, 52, 54, 55, 58–67, 69–72, 74, 75, 77, 80, 84]	71%	59
General [1, 5, 8, 14, 15, 25, 26, 47, 50, 51, 53, 56, 57, 78, 79, 81, 82, 85–91]	29%	24
**Country**		
Developing[19, 20, 22, 23, 29, 30, 39, 42, 50, 51, 54, 64, 78, 80–82, 84, 85]	22%	18
Developed [12–18, 21, 24–28, 31–38, 40, 41, 43–49, 52, 53, 55–63, 65–77, 79, 83]	67%	56
General [1, 5, 8, 86–91]	11%	9

Fifty-six out of the 83 studies (67%) were conducted in developed countries and 59 0f the 83(71%) were focused on a specific flood event. Regarding the demographics of the papers, six (7%) focused on children and adolescents, forty-five (54%) on adults, and 4 (5%) on older adults (see Table 1).

### Main outcomes

**Fig 2:**
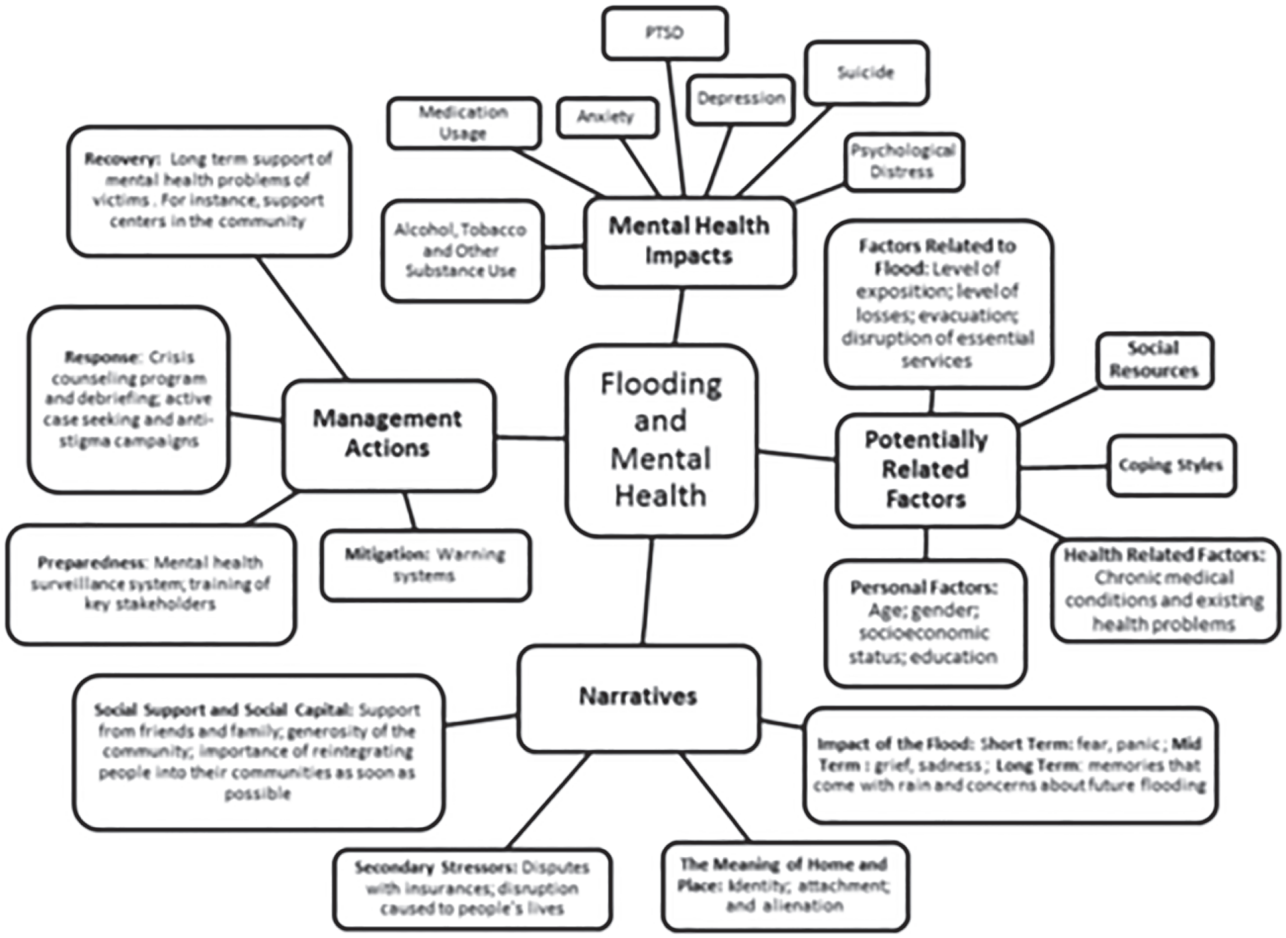
Conceptual Map.

Fig. 2 shows the conceptual map constructed after the mapping review, including the main aspects that have been researched in relation to floods and mental health.

#### Mental Health Dimensions

The mental health dimensions studied were as follows.

- **Post-traumatic stress disorder (PTSD)**: There was a significant increase in cases of PTSD in the affected areas, when compared with PTSD prevalence before the flood, assessed retrospectively [12]. Studies comparing affected and non-affected areas indicated a higher prevalence of PTSD or PTSD-related symptomatology in the flooded area [13–17]. It has been suggested that PTSD, or symptoms associated with PTSD, could be responsible for the development of other mental health problems such as depression, anxiety or substance abuse disorders [18]. Among the 29 cross-sectional studies without a baseline or a control group, 24 (83%) assessed PTSD or undiagnosed PTSD but showing symptoms of PTS [18–41]. As might be expected, different questionnaires were used to assess PTSD. The most commonly used questionnaires were the Impact Event Scale (IES) and the Post Traumatic Stress Disorder-civilian checklist (PCL-C).
- **Anxiety:** Groups exposed to flooding events showed higher levels of anxiety when compared with the non-exposed groups [14, 15, 17, 42]. Anxiety measures were included in eight out of the 29 (27.5%) cross-sectional studies [18, 24, 26, 27, 31, 32, 34, 40]. These included the Beck Anxiety Inventory (BAI), the Geriatric Anxiety Inventory, the Hopkins Symptom Checklist-25 and the GAD-7.
- **Depression**: Overall, people from the flood-affected areas experienced an increase in depression symptomatology [12, 43, 44]. This pattern was also found in children [45]. People in flooded areas were also more depressed than those in non-affected zones [15, 17, 42, 46], although this was not found to be the case in an Australian study conducted with older adults [14]. In the USA, an analysis of a subsample of the older-old (> 70 years old] people revealed similar results: the level of exposure was not associated with depression after the flood [44]. Thirteen out of the 29 cross-sectional studies (45%) included a measure of depression [18, 19, 24, 26, 27, 30–32, 34, 37, 39–41]. The questionnaires used to assess depression included the Centre for Epidemiological Studies Depression Scale (CES-D), the Hopkins Symptom Checklist-25 and the Beck Depression Inventory (BDI).
- **Suicide**: There was contradictory evidence regarding suicide following a flood event. A study in the USA reported an increase in the rates of suicide following a flood event [47], but this was not replicated in Australia [48]. The Australian study did not find differences in the suicide rates between flooded and non-flooded areas. These studies used administrative data in order to assess the outcome. Another study carried out in the USA [49] reported that greater amounts of time spent volunteering in flood recovery efforts were associated with increased feelings of belonging and a decrease suicide ideation that is an increase in desire to take one's own life or in thinking about suicide without actually making plans to commit suicide. This may explain differences in suicide patterns.
- **Psychological Wellbeing/ Psychosocial distress:** Overall psychological health and mental health-related quality of life was significantly worse in affected areas compared to non-affected areas [13, 16, 17, 33, 50–53]. An exception was the Australian study conducted by Bei and colleagues [14]. Nine out of the 29 cross-sectional studies (31%) also included some measure of general psychological wellbeing [19, 20, 26–28, 37, 54–56]. The most common measures to evaluate the psychological distress were the General Health Questionnaire (GHQ-12], the Health Related Quality of Life Short Form 12 (SF-12) and the World Health Organisation’s instrument—WHO-5.
- **Increase in tobacco, alcohol and other substance use:** One Australian paper [57] reported an increased use of tobacco and alcohol (self-reported], although this was not found in a USA study where the levels of use remained the same [12], nor in Canada [16] when comparing cases with controls. However, Peek-Asa [28], in a study carried out in the USA, found that alcohol abuse in males was associated with flood-related job loss or disruption.
- **Increase in medication usage:** In France, using administrative data, there was an increase in the number of psychotropic drug prescriptions during the three weeks following flooding and this was detected at a higher rate in females [58]. Similarly, using self-reported data, an increase in medication was also found in Australia [57] and in Canada [17], although no differences between cases and controls were found in another Canadian study [16].

In this section we present a number of factors that previous work has reported to be associated with the mental health status of people affected by floods. To summarise, we did not distinguish between factors considered to be confounders from mediators or moderators of the association between flooding and mental health, except where previous work has labelled them in such a way.

#### Factors related to the flood

The higher the level of exposure (measured in most of the cases as a combined index of different aspects related to the level of losses/damage and the threat/harm] the higher levels of mental health-related problems were reported, particularly PTSD [21–23, 26, 28, 34–38, 40, 41, 43–45, 54, 59, 60]. Similarly, flood level in the house was associated with an increase in the levels of mental problems [15, 27, 52, 53], although this relationship was not found in children [40]. Those who were evacuated experienced higher psychiatric symptomatology [14, 24, 27, 40, 53]. The inability to collect possessions [20] and the perceived severity of loss [20] and threat [32] were other factors associated with an increase in mental health-related problems. With respect to the type of flood, Liu et al [22] found that compared with sustained rainfall in a river catchment leading to the flood, victims of flash floods have an increased risk of developing PTSD. Those who suffered financial losses as a consequence of the flood [14, 27], problems with insurers [53], a high level of disruption to daily routines -including temporary or permanent loss of employment [14, 31, 53, 61] or loss of services [20, 27]—presented with higher levels of mental health problems.

#### Social resources

Different studies have assessed the link between social support and mental distress. In general, these studies found an inverse association [14, 32, 33, 37, 46, 62]. However, the relationship between social support and mental distress is more complex. For instance, Norris et al (2004) [59] found that six to 12 months after the impact, social support was a protective factor for mental disorders. At the midpoints of the four waves of their study (12 and 18 months), both processes (i.e. social support and distress) emerged as causal paths. Finally, in the last phase of the assessment (18 and 24 months), it seems that the social selection mechanisms explained the results (i.e. those with PTSD had less social support). On the other hand, parental support was a protective factor in children and adolescents [36]. Conversely, family conflict and overprotectiveness were associated with an increased risk of developing PTSD symptomatology [36], as well as a need for relationship adjustment because of a reduction in the level of relationship consensus, cohesion and satisfaction. [25, 35].

#### Coping factors

Several studies have addressed the impact of coping styles in the development of mental disorders after a flood, showing that positive and proactive behaviours were associated with positive mental wellbeing [13, 14, 31–33, 42, 63].

#### Health-related factors

Poor mental health status before the flood [43] [44, 45], as well as existing physical health problems [13, 24, 27, 53], were associated with post-event mental health. With regard to children, Peng et al (2001][29] found that pre-flood behavioural characteristics (assessed retrospectively] were associated with PTSD. Examples of pre-flood behavioural characteristics include children having difficulty sitting quietly for a long time, hyperactivity and restlessness.

#### Personal factors

There is contradictory evidence regarding the effect of gender and age. While some papers found poorer mental health in females following floods [21, 22, 26, 27, 53], others found no relationship [13, 14, 17, 29, 31, 32, 37, 42, 43]. Conversely, Peek-Asa [28] found that males had more alcohol-related problems. With regard to age, some authors found a protective effect of older age [21, 43, 44, 53], whilst others found an increase in the risk for mental disorders in older adults [22, 30, 37]. Finally, other papers have not found any such relationship [13, 14, 27, 31, 32, 42]. Socioeconomic status has been systematically associated with poor mental health outcomes after exposure to floods as the lower the socioeconomic status, the higher the risk of mental health problems [20, 41, 43, 46, 53, 55].

Only a few papers have addressed the medium- or long-term impact of flood events on mental health. The percentage of respondents who had a GHQ score GHQ greater than 2 decreased substantially over time but then slightly increased around the first anniversary of a flood in Thailand [64]. A GHQ score greater than 2 indicates satisfactory current mental health. Two years after a flood in Canada, Maltais and collaborators[17] found that the people affected presented higher levels of depression, PTSD symptoms, psychological distress and poorer adjustment. Similar results were found in Mexico, whilst rates of PTSD and depression declined over the two years, the prevalence remained higher than the overall rate for Mexico [46, 59] and the USA [12]. A significant reduction in health-related quality of life was found in South Korea 18 months after of the flood [65]. However, the mental health component of the quality of life instrument did not change [65]. Four years after the flood, current PTSD and depression scores were still associated with the level of flood exposure in France [34]. This result was sustained five years after the flood [38]. High rates of PTSD were found in children two years after flooding in the USA [21, 36] and in China [29].

Qualitative studies give a voice to flood survivors, helping us to understand the subjective experience and how flooding affected a person’s mental health Results from qualitative studies paralleled those reported from quantitative designs. The following main topics emerged: a) Impact of dangers posed by floods on mental health [66–71]; b) secondary stressors, such as disputes with insurance and construction companies, problems with personal relationships (at home, work and with friends), lack of understanding, economic problems and problems with employment, that ultimately led to mental health problems [66–68, 70–73], and loss of confidence in authorities[73]; c) memories that come with rain and concerns about future flooding [66–69, 73]; d) The long-term impact of floods on mental health [67, 70, 73, 74]; and e) The meaning of home and place, and how people can be defined by the place where they live, and the memories associated with this place. (Losing one’s home and other personal possessions, as well as the resulting displacement had a strong impact on their mental health) [66, 68, 72–75].

Some qualitative studies also describe what help was given to victims of floods to assist with their recovery. Interviewees cited social support from friends and family, along with generosity and altruism of the community immediately after the flood [68, 75]; good emergency response mechanisms [68, 71]; and the importance of creating safe spaces within the school classroom to explore victims’ flood experiences [69]. Other findings suggested reintegrating people into their communities as soon as possible; reducing the waiting lists to access health and social services; simplifying the insurance-related process [67, 69, 71]; and providing long-term mental health support [71, 74].

Several papers included suggestions for management actions to reduce the mental health impacts of floods. Regarding preventive or mitigation strategies, just one study illustrated the impact of warnings on subsequent mental health, suggesting that longer warning times had a small but significant effect in protecting mental health [53, 56]. The other studies did not provide information on the effectiveness of the strategy. However, they described some of the actions undertaken to reduce the mental health impact of floods. These strategies were related to a) preparedness: such as including mental health problems in the health surveillance system [76, 77]; and mental health specialists in the national disaster risk management committee [78]; as well as with b) acute response and recovery, with actions related to the identification and provision of immediately needed health services. Further strategies for the provision of medium- and long-term responses for persons with post-flood mental health problems include: both targeting psychological support and providing help with financial matters or assistance with housing[79]; and the development of mental health community centres with an strong focus on community activities, including anti-stigma campaigns related to mental disorders, as some people may be afraid to acknowledge they suffered emotional problems [78, 80–82]. Victims of disasters seek support from trusted members of their own communities rather than from mental health professionals [78, 81, 83]. So the role of NGOs and other stakeholders (such as religious leaders and teachers] in covering psychosocial needs after a flood in developing countries [78, 82, 84, 85] and in developed ones [83] is important. Hence, it is necessary to train them and to tailor the responses to the local context [82, 83, 85]. The training of people responsible for access to and provision of services in flood prone areas can be seen as a social capital preparedness strategy, related to the capacity to respond and the resilience of the community. Further, studies suggest there is a need to implement a stepped-care model. This is a model where the least intensive but likely to be effective intervention is used by first trying one intervention and either stepping up or stepping down the intensity dependent on the response. This type of model actively seeks people suffering mental disorders at the community level, and is conducted by social workers and/or lay people. The mild cases would be treated in primary care and the severe cases in specialised care. This highlights that the majority of the reactions to flooding are normal [40, 80, 83, 84]. Lastly, it is also important to provide public health advice through regular media releases [76] and to set up and monitor a health (including mental health) hotline [76, 77].

Eleven literature reviews are also included in this systematic mapping review. Only two were focused on the impact of floods on mental health [5, 81], With regard to the study conducted by Stanke et al [2012], 48 papers were included of which 30 assessed people's mental health after an extreme event [hurricanes, typhoons, or tsunamis] and 18 assessed people’s mental health after floods (37.5%). The majority of these 18 studies were conducted with adults. The main conclusion of this review was that flooding was a stressful experience, and that the stress continues for a long time after the water has receded. Floods exacerbate, precipitate or provoke mental health problems. The review conducted by Bhamani et al (2012) is a narrative review that included only 13 papers. Their conclusions are very similar to those of Stanke and colleagues, although they focused on low- and medium-income countries. Another review aimed to investigate the impact of natural disasters on suicide [86]. The authors found three papers that had focused on floods and suicide. Only one of them was also identified in our research but this could be due to the different definitions of a flood. The authors conclude that evidence suggests an increase in both suicidal ideation and attempted suicide in Chinese areas affected by periodic flooding and in young adults who were exposed to the Buffalo Creek floods when they were children. A recent review by Lowe [8] focused on the risk factors for mortality and morbidity pre, during and post a flood event. With regard to mental health, the authors concluded that during floods, females, elderly and children appear to be at greater risk of psychological and physical health effects. According to this review, other risk factors for mental health problems include previous flood experiences, greater flood depth or flood trauma, existing illnesses, low education or socio-economic status and social connectedness. Cherniack (2008) reviewed the impact of natural disasters on the elderly, and included a special section on floods [87]. The author reviewed the impact that floods had on mental health and concluded that there is contradictory evidence because, in some cases, older victims experienced more distress, while in other studies the oldest participants showed more resilience. Finally, six studies were included that aimed to review the impact that floods have on human health. All these reviews included a special section on mental health [1, 79, 88–91] and all concluded that mental health problems occurred directly because of the experience of being in a flood, or indirectly during the restoration process. However, these papers also pointed out that there was a lack of studies undertaken in vulnerable populations and that there were some methodological problems with those that had been conducted.

Fig. 2 presents these findings from the review including mental health impacts, potentially related factors, narratives and management actions. This provides a visual summary of the results.

## Discussion

To the best of our knowledge this is the first mapping review that focuses on the impact on mental health of fluvial flooding caused by heavy rain in the river catchments. We have excluded research reporting the mental health effects following typhoons, hurricanes and tsunami, and we provide a mixed approach including both qualitative and quantitative studies.

The inclusion of narratives is crucial for the new person-centred approach in public health [92]. This review suggests that floods have a potentially negative impact on mental health, with increasing levels of PTSD, anxiety, depression and use of psychotropic medication when assessed by means of administrative records. There is conflicting evidence with regard to suicide and tobacco, alcohol and substance abuse. The impact of flooding on mental health is similar in both developed and developing countries. However, there could be cultural differences related to the expression of emotions. Although the level of exposure to floods has been systematically associated with mental health problems, the paucity of longitudinal studies and lack of attention to confounding factors precludes strong conclusions. For instance, low socioeconomic circumstances are linked to poor mental health, and the impact of floods on mental health is higher in areas of material deprivation. A lack of control for socioeconomic circumstances would, therefore, lead to biased estimates of the flood impact on mental health and, potentially, inappropriate decision making as a result. Therefore, there is a wider problem in the literature on flooding and mental health – a dearth of studies that address the potentially causal mechanisms related to socioeconomic status and, therefore, identifying appropriate mediators and confounders.

This is the first review that maps quantitative and qualitative studies. The results were triangulated and suggest that information was very similar. However, qualitative studies highlighted the importance of house and place as “identity” and the importance of reintegrating people as soon as possible back into their community. The secondary stressors associated with the flood (i.e. dealing with restoration and insurance companies) are important issues to investigate in future research. This was highlighted in a recent review dealing with secondary stressors and extreme events and disasters [8]. The long-term effects of floods on mental health could be explained by these facts and also by the degree and impact of the losses. The paper by Raguenaud and collaborators [77] presents the epidemiological surveillance of mental health services after the Xynthia storm in France, pointed out that peaks of new cases of poor mental health coincided with the period when the authorities determined which houses were inhabitable and with the period when the technical experts assess the value of these houses.

This review has summarised some responses given in the aftermath of a flood event. Similar to the narrative data, these case studies concluded that both acute responses and long-term support are needed to reduce the mental health impacts. It is also important to have a well-trained workforce dealing with mental-health related issues [93]. Geographic Information Systems (GIS) can also be useful in the identification of populations that will need more s resources, complementing information coming from other surveillance systems [50, 51, 93, 94]. Only two of the paper included in this review used GIS [50, 51]. More studies employing GIS-based strategies would enhance the scientific literature on the impact of flooding on mental health. An example of how GIS can be used to enhance scientific literature would be the provision of a map that highlights areas of increased use of mental health services corresponding to areas of flooding in a time-dependent manner. The advantage of maps is that they display complex spatial information in a rigorous and meaningful way that can be easily interpreted by researchers, practitioners and policymakers.

In this review we have found some important research gaps. Most of the quantitative studies employed cross-sectional studies without a control group. Additionally, most of these studies adjusted by level of exposure, however, it is important to note, that different definitions related to exposure were used. While some studies considered exposure as synonymous with living in the affected area by the flood (regardless of the personal experience), other papers defined exposure as being personally affected by the flood. The impact of a flood can be assessed as the level of water in the home; the level of danger posed to life; or the level of material and human losses. Although there have been some attempts to develop an all-encompassing definition of exposure that combines all these flood exposure-related issues [43–45, 60, 95] more research is needed in this topic. Few studies include baseline measures. Although we acknowledge that it is difficult to include a baseline measure, administrative health records could be used to overcome this problem. These datasets can give invaluable information relating to suicide rates, psychotropic medication use, and mental health–related services in the aftermath of a flood (e.g. emergency visits, hospitalisation due to psychiatric reasons) that can be compared with rates before the event. The use of consistent questionnaires across studies related to natural disasters in ongoing routine longitudinal studies could also help resolve this limitation.

There is a lack of studies that report on results from monitoring of the ongoing mental health of the population affected by the flood. Most of the studies were conducted during the first year, with no medium- to long-term follow-up. This is important because acute stress-related response, if not addressed properly, can evolve to more severe disorders such as PTDS and depression. It is especially important to monitor these two conditions due to the high suicide risk associated. Therefore, information about PTSD symptomatology, depression and risk of suicide should be routinely assessed. In addition, due to the recognised long-term impact on mental health, more studies are needed to understand the patterns associated with recovery and its related factors, especially to ascertain which factors make communities more resilient [96–99]. There is also a lack of studies using qualitative methods. In this mapping review, we have only identified ten studies and all were conducted in the UK, Canada or USA. The use of mixed methods enriches the conclusions, gaining a depth of understanding to the problem. However, the qualitative studies also had some limitations: there was a lack of discussion on the philosophical underpinnings of the study; on the sampling procedure (i.e. what type of sampling was used? was sampling done until saturation in data was reached?); on the role of the researcher and the relationship with participants; and, finally, on the identification of assumptions and biases of researchers. Unlike quantitative research, the belief system of the researcher impacts on the interpretation of the research and it is therefore important to discuss the philosophy underpinning the research.

Concerning responses, there is limited evidence regarding their effectiveness or its impact (monitoring of the system). Different reviews addressing the effectiveness of public health or mental health interventions to reduce the impact of climate change reached the same conclusions, that is, more effort is needed to evaluate the effectiveness of these interventions [100, 101].

Finally, we found a lack of information on vulnerable populations such as children, adolescents, people living with disabilities and the elderly. A systematic review on the impact of natural disasters on the elderly concluded that there is contradictory evidence regarding whether older individuals have a worse or more favourable psychological outcome than younger individuals [87]. Taking into account the increasing proportion of the population who are ageing in most countries, and the fact that, due to the climate change, floods are going to be more frequent [4], having evidence on the impact of flood on the mental wellbeing of the elderly is crucial for policy makers and practitioners.

Our systematic mapping review has a number of limitations. First, there are limitations relating to the selection criteria. Studies where the flood followed extreme events were excluded from the study. Therefore, potential studies focusing on floods, such as the extensive inundation of coastal land following the 11 March, 2011 tsunami in the Northeast of Japan, have not been included. However, our main aim was to focus on the most frequent types of flooding caused by rivers overflowing their banks or levees and not to be confounded by additional impacts that are not only due to flooding. Secondly, mental health outcomes are difficult to define. Although we included in our search strategy the most common disorders and key words associated with natural disasters such as depression, anxiety, PTSD, mental, and psychological, it is possible that we have missed some research papers that define other outcomes.

## Conclusions

Floods are the most common type of global natural disaster responsible for almost 53,000 deaths in the last decade. In March 2013, the Climate Change Institute at the Australian National University released a report highlighting the increasing likelihood of flood events so the magnitude of the problem will increase in the future. The impact of floods on those who experience them can be significant. In addition to economic loss, detrimental short-, medium- and long-term effects on wellbeing, relationships and physical and mental health are common. According to a UK study, mental health problems are responsible for more than 80% of all the estimated Disability Adjusted Life Years (DALYs) attributable to floods.

To the best of our knowledge this is the first mapping review that focuses on the impact on mental health of fluvial flooding caused by heavy rain in the river catchments. We have excluded research reporting the mental health effects following typhoons, hurricanes and tsunami. PUBMED and Web of Science were searched to identify all relevant articles from 1994 to May 2014. The search was restricted to the past 20 years because from the early 1990s there has been an increasing focus on empirical evidence-based research and there was also a change in the description of mental disorders in the 4^th^ version of the Diagnostic and Statistical Manual of Mental Disorders (1994). We used free text and words were restricted to title and abstract. The word “flood” has an asterix (*) added as a wildcard in order to pick up plurals and other words such as “flooding” using Boolean logic. The electronic search strategy identified 1331 potentially relevant papers. Finally, 83 papers met the inclusion criteria. Table 1 provided a summary of the characteristics of the included manuscripts. 65 per cent of the manuscripts used quantitative methods. Of these, 54 per cent were cross-sectional surveys without control groups. Only 23% of the cross-sectional surveys included a control group and only eight studies (15%) utilised a baseline measure. Ten (12%) were qualitative studies dealing with the subjective experiences of flood survivors. Eleven (13%) reviews dealing with the effects of flooding on mental health were also included. Only two reviews were specifically focused on the impact of floods on mental health. However, these also included all types of flood-related events (tsunamis, hurricanes, typhoons). Fifty-six out of the 83 studies (67%) were conducted in developed countries and 59 of the 83 (71%) were focused on a specific flood event.

Fig. 2 showed the conceptual map constructed after the mapping review, including the main aspects that have been researched in relation to floods and mental health. Four broad areas are identified: i) the main mental health disorders—post-traumatic stress disorder, depression and anxiety; ii) the factors associated with mental health among those affected by floods; iii) the narratives associated with flooding, which focuses on the long term impacts of flooding on mental health as a consequence of the secondary stressors; and iv) the management actions identified. The mental health dimensions studied were post-traumatic stress disorder (PTSD), anxiety, depression, suicide, psychological wellbeing and psychosocial distress, increase in tobacco, alcohol and other substance abuse, and an increase in medication usage. The quantitative and qualitative studies have consistent findings.

A number of research gaps can be teased out from this review. The inclusion of narratives is crucial for the new person-centred approach in public health. Although the level of exposure to floods has been systematically associated with mental health problems, the paucity of longitudinal studies and lack of attention to confounding factors precludes strong conclusions. For instance, low socioeconomic circumstances are linked to poor mental health, and the impact of floods on mental health is higher in areas of material deprivation. A lack of control for socioeconomic circumstances would, therefore, lead to biased estimates of the flood impact on mental health and, potentially, inappropriate decision making as a result. Therefore, there is a wider problem in the literature on flooding and mental health: a dearth of studies that address the potentially causal mechanisms related to socioeconomic status and, therefore, identifying appropriate mediators and confounders. Very few studies have used mixed methods to quantify the size of the mental health burden as well as exploration of in-depth narratives. Methodological limitations include control of potential confounders and short-term follow up.

Most of the quantitative studies employed cross-sectional studies without a control group. Additionally, most of these studies adjusted by level of exposure. Whilst some studies considered exposure as synonymous with living in the affected area by the flood [regardless of the personal experience], other papers defined exposure as being personally affected by the flood. Although there have been some attempts to develop an all-encompassing definition of exposure that combines all these flood exposure-related issues more research is needed in this topic. Few studies include baseline measures, although administrative health records could be used to overcome this problem. These datasets can give invaluable information relating to suicide rates, psychotropic medication use, and mental health–related services in the aftermath of a flood (e.g. emergency visits, hospitalisation due to psychiatric reasons) that can be compared with rates before the event. The use of consistent questionnaires across studies related to natural disasters in ongoing routine longitudinal studies could also help resolve this limitation.

This review has summarised responses given in the aftermath of a flood event. There is a lack of studies that report on results from monitoring of the ongoing mental health of the population affected by the flood. Most of the studies were conducted during the first year, with no medium- to long-term follow-up. This is important because acute stress-related response, if not addressed properly, can evolve to more severe disorders such as PTDS and depression. Therefore, information about PTSD symptomatology, depression and risk of suicide should be routinely assessed. In addition, due to the recognised long-term impact on mental health, more studies are needed to understand the patterns associated with recovery and its related factors, especially to ascertain which factors make communities more resilient. Similar to the narrative data, these case studies concluded that both acute responses and long-term support are needed to reduce the mental health impacts. It is also important to have a well-trained workforce dealing with mental-health related issues. More studies employing GIS-based strategies would enhance the scientific literature on the impact of flooding on mental health. The provision of a map can highlight areas of increased use of mental health services corresponding to areas of flooding in a time-dependent manner. The advantage of maps is that they display complex spatial information in a rigorous and meaningful way that can be easily interpreted by researchers, practitioners and policymakers.

Finally, we found a lack of information on vulnerable populations such as children, adolescents, people living with disabilities and the elderly. A systematic review on the impact of natural disasters on the elderly concluded that there is contradictory evidence regarding whether older individuals have a worse or more favourable psychological outcome than younger individuals. Taking into account the increasing proportion of the population who are ageing in most countries, and the fact that, due to the climate change, floods are going to be more frequent, having evidence on the impact of flood on the mental wellbeing of the elderly is crucial for policy makers and practitioners.

## Supporting Information

S1 PRISMA Checklist (PDF)

S1 Supporting Information (PDF)
