# Supplementary material for: Flooding and Mental Health: A Systematic Mapping Review
Source: PLoS One. 2015 Apr 10;10(4):e0119929. doi: 10.1371/journal.pone.0119929 (PMC4393088; doi:10.1371/journal.pone.0119929)
Supplement: S1 Supporting Information — (PDF) [file pone.0119929.s002.pdf]

S1. Supporting information. Summary of the papers included in the review.

Quantitative Studies

Ref	1st_Author, year and country	Aim	Age category (N)	Design	Control Group	Baseline	Severity of Exposure	Use of Administrative Data	Mental Health Dimensions	Main Results
**13**	Alderman (2013) - Australia	To assess the effects of the **2011 floods in Brisbane** on resident's physical and mental health	Adults (N=960)	cross-sectional	Yes	No	**No**	No	Kessler 6 scale for Psychological Distress; Post traumatic Stress Disorder-civilian checklist (PCL-C); The Groninger Sleep Quality Scale(GSQS)	People who reported direct flood impact had higher psychological distress; more problems with sleeping; and a higher probability of Post-Traumatic Stress Disorders (PTSD) (after adjusting by gender, age, employment, education, and the existence of health problems)
**19**	Apisarnthanarak (2013) Thailand	To evaluate hospital preparedness as well as to assess the psychological impact of floods among infection preventionists (IPs) in the initial 6-month interval after a severe flooding occurred in central Thailand during the period September–November 2011	Adults (N=101 hospitals)	cross-sectional	No	No	No	No	Depression and PTSD, no clear how they were assessed	Overall, at the initial 6-month interval, 20 (63%) of 32 lead IPs in the flood-affected hospitals complained of having some psychological consequence related to the floods (e.g., PTSD, depression, inability to concentrate, insomnia, and having difficulties with family relationships).
**64**	Assanangkornchai (2007) Thailand	To investigate changes in the prevalence of adverse stress reactions in different areas of Hat Yai during the year immediately following a major natural disaster—the 2000 flood.	Adults (N=590 in baseline; 254 at the last follow up)	Longitudinal (monitoring after the flood)	No	No	Yes	No	General mental health was assessed with the General Health Questionnaire (GHQ) and PTSD with the Impact Event Scale (IES).	The percentage of respondents who had a positive GHQ score (GHQ > 2) decreased substantially at Times 3 and 4 but then slightly increased at Time 5 (anniversary). Overall, the GHQ changes appeared to be unrelated to gender and age group; The patterns of change in the IES scores I were similar to the patterns of change in the GHQ
**20**	Assanarigkornchai (2004) Thailand	To assess the effect of a severe flood occurred on Hat Yai (November 2000) on the mental health of the community and investigated predictors of adverse emotional responses.	General (N=590)	cross-sectional	No	No	**Yes**	No	General mental health was assessed with the GHQ and PTSD with the IES	Factors associated to GHQ were: the ability to collect possessions, and the perceived severity of loss. Factors associated to IES were: severity of loss, number of days without electricity, and number of cars damaged. On the other hand, socioeconomic status and expectation on flood were negatively associated.
**16**	Auger (2000) Canada	To measure the prevalence of posttraumatic stress disorder and emotional distress among victims of the Saguenay flood (1996) compared with those who were not affected by the flood.	Adults (n=141)	cross-sectional	Yes	No	No	No	Diagnostic criteria for PTSD was measured using the Post-traumatic Stress Disorder Reaction Index and high scores on the Self-Reporting Questionnaire on emotional distress.	Prevalence of posttraumatic stress disorder in the study group was close to 20% (odds ratio [OR] 6.08; 95% confidence interval [CI] 1.63 to 22.64). Prevalence of emotional distress in the study group was 29% (OR 2.42; 95% CI 1.04 to 5.61). There was no statistical difference in the consumption of alcohol or psychotropic drugs.

Ref	1st_Author, year and country	Aim	Age category (N)	Design	Control Group	Baseline	Severity of Exposure	Use of Administrative Data	Mental Health Dimensions	Main Results
**15**	Azuma (2014) Japan	To evaluate the health status of residents and the environmental risk factors of housing after flooding in different Japanese areas.	Adults (N=379)	cross-sectional	Yes	No*	Yes	No	post-traumatic stress disorder (PTSD)/acute stress disorder (ASD), depression and anxiety were included in the questionnaire	After one week, depression and anxiety were higher in the exposed group. Rates of depression were higher also after 6 months. PTSD prevalence was higher after 6 months. Level of exposure was related to increase the risk of mental health problems.
**14**	Bei (2013) Australia	To examine the impact of floods on the mental and physical health of older adults and explored risk and protective factors.	Older Adults (N=421 pre flood; 274 at follow up)	longitudinal	Yes	Yes	Yes	No	The Impact of event scaled revised (IES-R); The Geriatric Anxiety Inventory (GAI); Centre for Epidemiological Studies Depression Scale (CES-D); SF-12; Satisfaction with life Scale;	Compared to those not personally affected, personally affected reported higher rates of PTSD, and a greater increase in anxiety scores post-flood. Depressive symptoms and self-reported health were not different. Greater flood exposure and lack of social support were risk factors for poorer mental health. Use of maladaptive coping was associated with greater deterioration of mental health after the flood. On the other hand, emotion focused coping was protective against such deterioration.
**54**	Bich (2011) Vietnam	To ascertain the vulnerability and health impacts of a devastating flood in Hanoi (last days of October and first week of November 2008) by identifying the differences in mortality, mobility between floods affected and non-affected household	General (N=871 households)	cross-sectional	No	No	Yes	No	They asked if any family member ever have psychological problems e.g. Stress, nervousness, anxiousness, sleeplessness? Having psychological problems before the heavy rain/flood? Did the psychological problems get worse?	There were significant differences between the respondent form severe affected areas and those less affected. In less affected areas around 90% of those who ever had suffered psychological problems reported to have suffered before the flooding, while in the most affected areas were 72% and 50%. Only 33% of those in less affected areas said that their symptoms got worse, in comparison with 84% and 63% of people on more affected areas.
**36**	Bokszczanin (2008) Poland	To evaluate the role of family factors as predictors of PTSD symptoms among adolescents (28 months after the flood occurred in south-western Poland in June 1997).	Children and Adolescent (N=533)	cross-sectional	No	No	Yes	No	Authors used the Revised Version Mississippi PTSD scale	The extent of trauma exposure was correlated with PTSD, after 2 years of the event. PTSD was associated to exposure to floods fret adjusting by age, gender, place of residence, parental support, family conflict, and overprotectiveness. Family factors were strongly associated to PTSD
**21**	Bokszczanin (2007) Poland	To examine the prevalence and predictors of PTSD in a sample of students exposed to the flood occurred in south-western Poland in June 1,997	Children and Adolescent (N=239)	cross-sectional	No	No	Yes	No	Revised Civilian Mississippi PTSD Scale	17.7% adolescents met the criteria for PTSD. Level of trauma was the stronger predictor of PTSD. It was a triple interaction between exposure, gender and age. Girls and older boys exposed presented higher levels of PTSD symptoms. Exposed younger boys, however, do not present higher symptoms than non-exposed.

Ref	1st_Author, year and country	Aim	Age category (N)	Design	Control Group	Baseline	Severity of Exposure	Use of Administrative Data	Mental Health Dimensions	Main Results
**55**	Clemens (2013) Australia	To assess the population prevalence of property, income and emotional impacts of the floods, after the 2010-2011 floods of Queensland	Adults (N=6104)	cross-sectional	No	No	No	No	Queensland Self-reported Health Status (SRHS) They asked participants if they: (i) felt terrified, helpless or hopeless; (ii) Still currently distressed (between 2 and 5 months after)	One in seven (14.35%) felt terrified helpless or hopeless after the disasters. This prevalence was higher in women, among those in the most disadvantage area, working -age people, and those living in remote areas. Around 7% of respondents were still currently distressed. This was common among women and those living in remote areas.
**41**	Collins (2013) USA	To assess the health impacts of the 2006 flood	General (N=475)	cross-sectional	No	No	Yes	No	They asked participants: did you or other members of your household experienced 1 or more mental health problems during or in the first 4 months after 2006 floods? (they asked depression and PTSD)	18.3% answered yes to those questions. 17.5% experienced reporting depression and 8.6 PTSD. The following variables increased the odds of any mental disorder: adverse event experience, older age, financial difficulties, not a US citizen. On the other hand, having access to medical care, foreign-birth and lack of English proficiency were associated with decreasedodds of mental health problems.
**48**	De Leo (2013) Australia	This study compared the prevalence and characteristics of suicides following the January 2011 Queensland floods to the 11 years prior (for the period January–June) for two severely affected locations: Ipswich and Toowoomba.	General *Sample not stated*	longitudinal	No	Yes	No	Yes	suicide deaths in the register	Findings showed no significant increase in suicide rates during the 6 months after the floods.
**40**	Kreuger (2008) USA	To evaluate the effects of floods on the mental health of child and adolescents after the Great Flood in 1993 (Mississippi) and to propose an intervention model	Child and Adolescent (N=3876)	cross-sectional	No	No	Yes	No	the Revised Children's Manifest Anxiety Scale (RCMAS), and the Children's Depression Inventory (CDI).	Level of impact, damage to family and having been evacuated were associated with higher distress and mental health problems. The authors also proposed a model.
**45**	Felton (2013) USA	The current longitudinal study tested hypotheses about Nolen-Houseman's (1987, 1991) response styles theory (RST) of depression in a sample of child and adolescent public school students, exposed to the 2010 flood in Tennessee	Child and Adolescent (N=239 at wave 1; 227 at wave 2)	longitudinal	No	Yes	Yes	No	Mental health was assessed with the Children's Depression Inventory	Post flood depressive symptoms correlated positively with preload depression, preload rumination, post flood rumination, and the severity of one's flood-related experience—and correlated negatively with preload distraction. After controlling for preload depression, severity of one's flood-related experience and post flood rumination remained significant predictors of post flood depressive symptoms. Rumination on post flood depressive symptoms via the intervening variable of post flood rumination, and partial mediation of the effect of preload depression on post flood depression. Further, the interaction of rumination with flood-related stressors was moderated by age, suggesting that rumination may not become a strong cognitive diathesis for depression until adolescence

Author 17/8/2014 10:33 PM
Comment [1]: What does this term mean?

Ref	1st_Author, year and country	Aim	Age category (N)	Design	Control Group	Baseline	Severity of Exposure	Use of Administrative Data	Mental Health Dimensions	Main Results
**35**	Fredman (2010) USA	to test a model based on a social causation hypothesis of intimate relationship adjustment in post disaster PTSD symptoms, after the 1993 Missouri floods	Adults (N=250 women)	cross-sectional	No	No	Yes	No	Posttraumatic stress disorder symptoms were assessed with a modified version of the National Women's Study PTSD module	Results from structural equation modelling revealed a positive and significant direct association between threat/harm and PTSD symptoms. Conversely, loss was not significantly associated with PTSD symptoms, but was positively and significantly associated with relationship adjustment. Relationship adjustment was negatively and significantly related to PTSD symptoms
**43**	Ginexi (2000) USA	To explore the impact of the 1993 Midwest Floods on depression	Adults (N=893)	longitudinal	No	Yes	Yes	No	Centre for Epidemiological Studies Depression (CESD)	Predictors of depression symptomatology post disaster were: pre disaster symptoms, flood impact level, age (inverse association), household income, being separated (vs being married). There were interactions between: level of exposure and gender (impacted male tends to have higher symptoms); level of exposure and income, respondents with the lowest incomes had greater increases in depressive symptoms as a function of impact level; and level? of community and size of the community. The increase in depressive symptoms as function of flood impact was higher in small towns. When tested, the predictors of a diagnosis of depression, the risk factors were: previous diagnosis of depression, flood impact level, aged (protective) and household income.
**49**	Gordon (2011) USA	To test if the more that individuals actively engaged in community volunteering efforts (i.e., pulling together), the lower their levels of thwarted belongingness and perceived burdensomeness would be following the 2009 flood in Fargo, North Dakota	Young adults (university students) (n=210)	cross-sectional	No	No	No	No	Acquired Capability for Suicide Scale ACSS is a 20-item measure designed to assess one's fearlessness about the act of suicide	Greater number of hours spent volunteering was associated with ?lower levels of acquired capability for suicide at a trend level (i.e. increased feelings of belongingness and decreased feelings of burdensomeness)
**50**	Gruebner (2011) Bangladesh	To study self-related mental health status in several slums of Dhaka, by accounting for neighbourhood social and physical associations (including risk of flooding) using spatial statistics	General (N=1938)	cross-sectional	Yes	No	Yes	No	Authors used WHO-5 as a measure of self-rated mental health status.	Flood non-affectedness, defined as a composite measure including longer distances to the nearest river, whether the area was regarded as flood non-affected, and whether the area had an adequate drainage system, was positively associated with good mental health in some of the slums.
**51**	Gruebner (2012) Bangladesh	To study, by using geo-epidemiological approach, which factors contribute to the mental well-being in the slums of Dhaka	General (N=1938)	cross-sectional	Yes	No	Yes	No	Authors used WHO-5 as a measure of self-rated mental health status.	Flood non-affectedness, defined as a composite measure including longer distances to the nearest river, whether the area was regarded as flood non affected, and whether the area had an adequate drainage system, was positive associated with good mental health
**65**	Heo (2008) South Korea	To examine changes in general health status, the prevalence of depression and post-traumatic stress disorder (PTSD) symptoms, and the existence of pre-trauma contributing factors in an agricultural population following a massive flood occurred in 2006.	Adults (N=58)	Longitudinal	No	Yes	No	No	The authors had a baseline measure for SF-36. The mental health dimension was assessed 18 months after the event. Beck Depression Inventory (BDI) and PTSD domain of the Minnesota Multiphasic Personality Inventory(MMPIPTSD) and the Korean version of the Impact of Event Scale (IES-R)	SF-36- K decreased significantly, with the exception of the MH subscale. 31% of the subjects scored 24 or higher on the IES-R, which is the cut-off for a clinical diagnosing of PTSD. A total of 43% scored 17 or higher in the MMPI-PTSD scale. 22.41% qualified for PTSD in both instruments. With regard depression, 17.24% scored > 23 in BDI (severe depression)

Ref	1st_Author, year and country	Aim	Age category (N)	Design	Control Group	Baseline	Severity of Exposure	Use of Administrative Data	Mental Health Dimensions	Main Results
**39**	Ishikawa (2013) India	To examine the impacts of this disaster, one month after the August 2010 flood in Northern India	Adults (N=318)	cross-sectional	No	No	No	No	Depression was evaluated with the Patient Health Questionnaire (PHQ-2 items). Positive cases were interviewed by a psychiatrist	26 out 318 participants (8.17%) showed positive PHQ-2 items and were interviewed by the psychiatrists. Only 2 cases presented PTSD (0.006%)
**46**	Jones (2011) Mexico	To assess depression two years after the 1999 floods in Mexico; and to understand the relationship with socioeconomic status and inequality.	Adults (N=2750)	cross-sectional	Yes	No	Yes	No	Depression was evaluated with the CES-D.	Socioeconomic status, social embededness and city of residence (proxy of exposure) were predictors of depression. However, if city variable was recorded into three groups according to level of exposure (low, moderate, high); exposure did not contribute to explain the effect on recent depression.
**62**	Kanisaty (2008) Mexico	To examine social causation and social selection explanations for the association between perceptions of social support and psychological distress, after the October 1999 floods	Adults (N=557)	Longitudinal (monitoring after the flood)	No	No	Yes	No	Current (past 6-month) symptoms of PTSD were measured by using a modified version of Module K of Version 2.1 of the Composite International Diagnostic Interview (CIDI)	Structural equation modelling analyses indicated that social causation (more social support leading to less posttraumatic stress disorder [PTSD]) explained the support-to-distress relationship in the earlier post disaster phase, 6 to 12 months after the impact. Both causal mechanisms emerged as significant paths in the midpoint of the study (12 and 18 months). Only social selection (more PTSD leading to less social support) accounted for the support-to-distress relationship at 18 to 24 months after the event.
**47**	Krug (1999) USA	To determine whether natural disasters affect suicide rates, in overall and by type of disaster	General (N=377 counties)	longitudinal	Yes	Yes	No	Yes	County-specific monthly numbers of suicides from 1979 to 1993 were obtained from the National Centre for Health Statistics Multiple Cause of Death Data File.	Before floods, the suicide rate for the 308 affected counties (12.1 per 100,000 populations) was similar to that for the United States as a whole (12.3 per 100,000 populations). After floods, there was an increase of 13.8 percent (95 percent confidence interval, 6.1 to 22.1 percent; P 0.001) in the suicide rate in the counties affected, whereas the rate for the United States as a whole remained stable. As compared with the pooled predicate rate, there were significant increases in suicide rates, ranging from 9.1 to 24.3 percent, in each of the four post flood years , with the highest rate occurring in the fourth year after the disaster
**22**	Liu (2006) China	To estimate the occurrence and to assess the determinants of posttraumatic stress disorder (PTSD) in flood victims of the severe flood in the Hunan province in 1998 and 1999	GENERAL (N=33340)	cross-sectional	No	No	Yes	No	Diagnosis of PTSD made according to the DSM-IV	Among the interviewed participants, 8.6% met the diagnostic criteria for PTSD. Factors associated to PTSD were: being a women, age (older adults had higher risk compared those less than 18 years old); the flood type: and the flood severity.
**23**	Maghrabi (2012) Kingdom of Saudi Arabia	Exploring the impact of the floods occurred during 2009 on the mental health of the residents in the Eastern region of Jeddah Governorate	Adults (N=450)	cross-sectional	No	No	Yes	No	PTSD was assessed with PTSS-10	PTSS-10 scores were higher in men than in women and among those with a job (without adjusting). Scores were higher among those who witnessed the disaster, those who experienced physical injury, or loss or injury of one or more family member or friend. But it was not higher among those who lost their properties.
**38**	Maltais (2000) Canada	To compare 2 years after the floods in Sangueney (1996), the mental health of affected people and non-affected by the flood.	ADULTS (N=177)	cross-sectional	Yes	No	No	No	Mental health was assessed with the GHQ, 28 items version. The Beck Depression Inventory (BDI) , the Beck Anxiety Inventory (BAI), and the IES.	People affected reported higher scores in the 4 measures than people non affected, There were no differences according to gender.

Ref	1st_Author, year and country	Aim	Age category (N)	Design	Control Group	Baseline	Severity of Exposure	Use of Administrative Data	Mental Health Dimensions	Main Results
**37**	Maltais (2005) Canada	To review the factors associated to psychological distress two years after the floods in Sangueney (1996),	Adults (N=177)	cross-sectional	No	No	Yes	No	PTSD was evaluated with the IES scale; Depression was evaluated with the BDI; Psychological Health was evaluated with the GHQ-28.	Age, self-perceived stress during the flood, low social support and avoidance as a coping strategy were associated to PTSD. Depression was associated to having received less help than expected, low social support and the avoidance as coping mechanisms. Psychological distress was associated to having received less help than expected, and low social support
**24**	Mason (2010) UK	To examine the psychological impact of flooding	Adults (N=444)	cross-sectional	No	No	Yes	No	PTSD was assessed with the Harvard Trauma Questionnaire. The Hopkins Symptoms Checklist was used to assess anxiety and depression	After the flood, 27.9% of participants suffered PTSD. Around 24.5% anxieties and 35% depression. After adjusting by socio-demographic variables, having to vacate home after the flooding was associated to higher mental health problems. Coping mechanism were also associated to the different MH Problems
**18**	McMillen (2002) USA	This report empirically examines multiple explanations for the high rates of psychiatric comorbidity seen with posttraumatic stress disorder (PTSD), after the Midwest 1993 floods.	Adults (N=162)	cross-sectional	No	No	No	No	Subjects were interviewed about their psychiatric and social status with the Diagnostic Interview Schedule for DSM-III-R (DIS) and its Disaster Supplement.	Thirty-five subjects (23%) met criteria for PTSD related to the flood. PTSD was frequently comorbid with other disorders. Seventeen subjects (10%) developed a new, non-PTSD psychiatric disorder after the flood. New non-PTSD disorders were rare in the absence of PTSD symptoms. Though prior psychiatric history was predictive of developing PTSD, no support was found that prior psychiatric history contributed to PTSD through social vulnerability. Thus, support was found for a model in which PTSD contributes to the development of other disorders following trauma, whereas no evidence was found to suggest that comorbid disorders develop independently of PTSD following trauma
**25**	Monson (2009) USA	To examine the role of wives' relationship adjustment in testing associations between PTSD symptoms after the floods in 1993, and their world assumptions.	ADULT (N=58 couples)	cross-sectional	No	No	No	No	PTSD was assessed using the National Women's Study PTSD module	Neither husbands' nor wives' benevolent world assumptions predicted wives' PTSD symptoms. However, when husbands held less benevolent world assumptions there was a negative relationship between wives' assumptions and PTSD.
**58**	Motreff (2013) France	To estimate the short-term mental health impact of the February 2010 floods in terms of psychotropic drug delivery, on the resident population of the 15 coastal municipalities severely hit by the flooding	ADULT (N=20981)	Longitudinal	No	Yes	No	Yes	New Psychotropic treatments were obtained from the French National Health Insurance Inter-Regime Information System	The daily average number of new psychotropic treatment was 25, while the daily average in the 21 days following the storm was 43. The RR of new psychotropic drug treatments for both genders was 1.53 (1.39-1.61), being 1.64 (1.48-1.74) for women and 1.41 (1.23-1.54 for men). By type of psychotropic, tranquilizers (N05C) had a RR 1.78 (95% 1.59-1.89); hypnotics (N05B) had a RR of 1.53 (1.31-1.67); and antidepressant (N06A) had a RR of 1.26 (1.06-1.04). Women had an increase in the three types, while men only in tranquilizers.
**59**	Norris (2004) Mexico	To examine the stability of PTSD symptoms over four waves of a panel study conducted after the Mexican flood of 1999.	Adults (N=561)	Longitudinal (monitoring after the flood)	No	No	Yes	No	PTSD was measured by using a modified version of Module K of Version 2.1 of the Composite International Diagnostic Interview (CIDI), developed and translated into Spanish by the World Health Organization. They also assessed comorbid Major Depression	By the end of the study, rates of current PTSD had declined from 14 to 8% in Villahermosa and from 46 to 19% in Tezuitl´an. Even at 2 years post event, however, the prevalence of PTSD remained high enough to be of public health concern and much higher than the base-rate of current PTSD in Mexico (2%).

Ref	1st_Author, year and country	Aim	Age category (N)	Design	Control Group	Baseline	Severity of Exposure	Use of Administrative Data	Mental Health Dimensions	Main Results
**12**	North (2004) USA		ADULTS (N=162)	Longitudinal (monitoring after the flood)	No	No	No	No	Index and follow-up interviews used the DSM-III-R Diagnostic Interview Schedule/Disaster Supplement (DISIII-R) and included sections covering seven disorders: PTSD, major depression, panic disorder, generalized anxiety disorder, somatization disorder, alcohol use disorder, and drug use disorder.	New cases of PTSD flood related were 16% and 8% for Depression. No new cases of alcohol or substance abuse were detected.
**26**	Otto (2006) Germany	To investigate the influence of the belief in a just world (BJW) on important dimensions of mental health and psychopathology (e.g., depression), after the August 2002 floods in Saxon.	Adults (N=112)	cross-sectional	No	No	Yes	No	PTSD was assessed with the German version of the Impact of Event Scale (IES-R); Depression and anxiety were studied with the German version of Beck Depression Inventory and beck's Anxiety Inventory (BDI and BAI, respectively). General psychological symptoms were assessed with the German version of the Brief Symptom Inventory (BSI)	Personal Beliefs in a just world can be seen as a buffer, as it is associated to decrease the levels of anxiety, depression and general psychological symptoms (after adjusting by different factors such as level of losses and exposure, or sex). However, belief in a just world is not associated to PTSD.
**27**	Paranjothy (2011) UK	To assess the prevalence of and risk factors for the psychosocial consequences of the 2007 flooding	General (N=2266)	cross-sectional	No	No	Yes	No	Psychological distress was assessed with the GHQ-12; Anxiety with the GAD-7; Depression with the PHQ-9; and PTSD with the Post traumatic stress disorders checklist/	After adjustment, Psychological distress, anxiety, depression and probable PTSD were more probable among those affected than those non affected. All mental outcomes were associated with the perception that the finances will be worse. Disruption to essential services was also associated to all the mental health measures. Evacuation was associated with psychological distress and anxiety. Women presented higher odds than men to present a Mental health problem. People with reported existing medical problems were more likely to report any mental health problem.
**56**	Parker (2007) UK	To review the economic benefits generated by flood damage savings by household that warning facilitates; health effects of flooding and flood warnings; and the effect of warnings on loss of life and physical injury	Adults (non stated)	cross-sectional	No	No	No	No	Mental Health was assessed with the GHQ-12 and with the PTTS	Proportions of mental health problems among those warned and not warned are similar in the bivariate analysis. However, in multivariate analysis warning lead time emerged as one of the factors that had an influence on mental health at the worst time of flooding as measured by the GHQ-12. A longer warning time has a small but significant effect in reducing "worst time" score. It also influences the "current" stress levels of flood victims
**28**	Peek-Asa (2012) USA	This study examines student psychological health following the large June 2008 flood at a university	Young adults (university students) (N=1231)	cross-sectional	No	No	Yes	No	Symptoms of PTSD were measured through the modified Child PTSD Symptom Scale	Controlling for gender, ethnicity, grade, and damage to the student's home, students reporting work disruption were more than four times more likely to report PTSD symptoms (95% CI, 2.5-8.2).
**29**	Peng (2011) China	To explore the relationship between posttraumatic stress disorder (PTSD) and preload behavioural characteristics among children aged 7-15 years in Hunan, China, after the 1998 floods	Child and Adolescent (n=7083)	cross-sectional	No	No	Yes	No	PTSD was assessed by trained psychologists using DSM-IV criteria. Parents answered a questionnaire on pre-flood behavioural characteristics	The overall prevalence of PTSD was 2.05%. After adjusting by potential confounders (age, sex, flood type, flood degree, waiting for rescue, observing a victim drawing, family members scattered by the flood, and delay in the reopening of the school) pre-flood behavioural problems were associated to increase the risk of PTSD

Ref	1st_Author, year and country	Aim	Age category (N)	Design	Control Group	Baseline	Severity of Exposure	Use of Administrative Data	Mental Health Dimensions	Main Results
**52**	Reacher (2004) UK	To investigate the health impacts of severe river flooding, occurred on 12 Oct 2000	Adults (N=351)	cross-sectional	Yes	No	Yes	No	Psychological distress was assessed with the GHQ-12	Psychological distress (> 3 points at the GHQ-12) showed a four-fold higher risk in flooded compared to non-flooded subjects and a highly significant increase in risk with flood depth.
**60**	Smith (USA) 1996	To examine the relationship between coping and outcomes following the 1993 Midwest flood.	Adults (N=209 at baseline; 131 at follow up)	Longitudinal (monitoring after the flood)	No	No	Yes	No	Psychological Health was evaluated with the GHQ-28.	Coping strategies predicted outcomes after controlling for demographic and flood exposure. Active coping was associated to less psychological distress, while avoidant coping was associated to greater psychological distress. Flood exposure was also associated to psychological distress.
**61**	Smith (USA) 2000	To test if Psychosocial resource loss as a mediator of the effects of the 1993 Midwest flood exposure on psychological distress and physical symptoms	Adults (N=209 at baseline; 131 at follow up	Longitudinal (monitoring after the flood)	No	No	Yes	No	Mental health was assessed with the GHQ-12	Flood exposure was associated to psychological distress after the flooding and to psychosocial resources lost. This was related to psychological distress 6 months after the flood
**63**	Smith (USA) 2000	This study examined the relationship between religious coping by church members and psychological and religious outcomes following the 1993 Midwest flood.	Adults (N=209 at baseline; 131 at follow up	Longitudinal (monitoring after the flood)	No	No	Yes	No	Mental health was assessed with the GHQ-12	Religious attributions and coping activities predicted psychological and religious outcomes at both 6 weeks and 6 months after controlling for flood exposure and demographics
**30**	Telles (2014) India	To screen survivors of the Bihar floods (2008) a month after the event to determine their scores in the PTSD and/or depression and to correlate these scores with age and gender	General (N=1289)	cross-sectional	No	No	No	No	The Screening Questionnaire for Disaster Mental Health (SQD) was used to screen for PTSD and depression	People over the age of 60 years had higher scores for PTSD and depression than those in the younger groups.
**31**	Tobin (1996) USA	To predict risk factors associated to the development of PTSD, after a flood occurred in 1993	Adults (N=106)	cross-sectional	No	No	No	No	The Diagnostic Interview Schedule was adapted to screen for symptoms of anxiety and depression. Questions for PTSD were selected and modified form the University of Michigan version of the CIDI	With regard anxiety, 76% of the respondents indicated they had at least one anxiety symptom following the flooding, and 27% had four or more symptoms. Sixty-six per cent of respondents recorded at least one sign of depression and 50% had multiple depressive symptoms. Regarding PTSD, 71% of respondents showed symptoms following the flood. Factors associated to stress were: previous health conditions (in particular anxiety); employments status, and propensity to interpret flood negatively.

Ref	1st_Author, year and country	Aim	Age category (N)	Design	Control Group	Baseline	Severity of Exposure	Use of Administrative Data	Mental Health Dimensions	Main Results
**53**	Tunstall (2006) England and Wales	The study aimed: to establish the nature and extent of short and long term physical and psychological health effects of flooding on flooded residents in England and Wales; to examine whether the health of flood victims was worse than that of non-flooded residents in flood risk areas and also than that of the general population; to examine the factors that influenced the health outcomes of flood victims.	Adults (N=1510)	cross-sectional	Yes	No	Yes	No	General mental health was assessed with the GHQ and PTSD with the Post Traumatic Stress Scale	64% of the flood victims had a worst time score of 4 or more, conventionally taken as indicative of psychological distress, compared with 25% with this score at the time of the interview. The following factors were associated to GHQ at the worst time: problems with insurers, gender, prior health, uninsured losses, evaluation, time to get to normal, contaminated of flood waters, rented accommodation, warning time and aged +65 (more age, less impact). With regard PTSD, the factors are associated were: problems with insurers, prior health, gender, evacuation, depth in cm in main rooms, warning time, time to get back to normal, vulnerable housing, contaminated of flood waters, and aged 65+.
**57**	Turner (2013) Australia	To study the relationship between direct exposure and increase in tobacco, alcohol and medication use after the floods occurred in November 2010 and January 2011	Adults (N=960)	cross-sectional	No	No	Yes	No	Questions related to increase on tobacco, alcohol and medication use	Flood impact was found to be significant risk factors for increased usage for all substances group.
**44**	Tyler (2000) USA	to examine the potential moderating effects of social support and age among older adults exposed to the 1993 flood	Older Adults (N=651)	Longitudinal	No	Yes	Yes	No	Depression was measured with the CES-D	Previous levels of depression and social support were associated to post flood depression in both the sample of young-old (55-69) and older-old (>=70). However, flood impact was found to be associated only in the younger sample.
**38**	Verger (2000) France	To study factors associated to psychological distress 5 years after the 1992 flood in Vaucluse	Adults (N=500)	cross-sectional	No	No	Yes	No	PTSD was evaluated with the QE-PTSD	Authors found a significant exposure-effect relationship between the level of exposure and PTSD symptoms. Higher PTSD was found in women, participants older than 35 years old, participants with a lower income, and those with a history of psychological problems.
**34**	Verger (1999) France	To assess the psychological impact of the 1992 flood in Vaucluse(feasibility analysis)	Adults (N=81)	cross-sectional	No	No	Yes	No	PTSD was evaluated with the QE-PTSD. Anxiety was evaluated with the Spielberg questionnaire, and depression with the BDI	PTSD and Depression were associated to flood, but not anxiety.
**32**	Wind (2011) UK	To explore the relationship between social capital and disaster mental health outcomes (PTSD, anxiety, and depression) in combination with individual factors (appraisal, coping behaviour, and social support), after the 2008 flood in Morpeth	Adults (N=236)	cross-sectional	No	No	Yes	No	Symptoms of anxiety and depression were assessed by the Hopkins Symptom Checklist-25. PTSD was evaluated with the PCL-C.	Cognitive social capital was associated to depression, anxiety and PTSD beyond the individual characteristics. Depression was also associated to individual social support and coping intensity; anxiety was related to primary appraisal, individual social support, coping intensity; and structural social capital; lastly, PTSD was also associated to coping intensity.
**33**	Wind (2012) UK	To test how social capital is related to post-disaster mental health (September 2008 flood in Morpeth)	Adults (N=232)	cross-sectional	No	No	Yes	No	PTSD was evaluated with the PCL-C.	The effect of structural social capital on PTSD was indirect via individual psychosocial variables. This association was mediated by Cognitive social capital and collective efficacy.

Ref	1st_Author, year and country	Aim	Age category (N)	Design	Control Group	Baseline	Severity of Exposure	Use of Administrative Data	Mental Health Dimensions	Main Results
**42**	Wind (2013) India	To examine the immediate impact of a recurrent flood on mental health and functioning among affected population, compared with a population in the same area non affected	Adults (N=626)	cross-sectional	Yes	No	No	No	Symptoms of anxiety and depression were assessed by the Hopkins Symptom Checklist-25 (HSCL-25). Psychological and physical functioning was assessed by using the Short Form-12 (SF-12).	The affected group scored significantly higher in Depression. Anxiety and lower in the Mental Component of the SF-12. In the affected group, authors did not find any association between socio-demographic characteristics and mental health.

Qualitative studies

Ref.	1st_Author, year and country	Aim	Age category (N)	Technique	Mental Dimension	Main Results
**66**	Carroll (2009) UK	To assess the health and social impacts of the 2005 floods in Carlisle (focus on identity and the meaning of home)	Adults (N=40)	Interview and focus groups	Subjective experiences of having lost homes, impact of displacement and destruction	Psychological stress people were suffering was related to the destruction and reconstruction of their homes. Home and possession are related to identity. When home is destroyed, their identity is, in some sense, broken.
**67**	Carroll (2010) UK	To assess the health and social impacts of the 2005 floods in Carlisle (focus on consequences)	Adults (N=40)	Interview and focus groups	Subjective experiences of distress and mental health related problems	Respondents spoke of mental health problems caused primary by the flood themselves (the danger), but also indirectly by the disputes with insurance and construction companies, which participants felts had caused and exacerbated mental health problems.
**69**	Convery (2010) UK	to assess the way the 2007 flood in Hull affected the way this affected schools and pupils in the city	Child and Adolescent (but the interviews were carried out with the teachers N=6)	Interviews	Narratives of how the floods impact children and adolescent and the role of schools in deal with it	The paper indicates the importance of reintegrating children into community structures (school) as arson as possible after the flood and of creating safe spaces to explore their floods experience
**68**	Convery (2008) UK	To assess the health and social impacts of the 2005 floods in Carlisle	Adults (N=16)	Interview and focus groups	Subjective experiences of distress and mental health related problems	Respondents spoke of mental health problems caused primary by the flood themselves (the danger), but also indirectly by the disputes with insurance and construction companies, which participants felts had caused and exacerbated mental health problems. They also explained what helped after the flood. The comment on the social support and the formal Reponses (especially emergency response mechanisms) and the generosity of the community.
**75**	Harvey (1995) USA	To investigate people's accounts of loss and recovery from the 1993 Midwest flood	Adults (N=45)	Interviews	Subjective experiences of recovery among people affected by the 1993 flooding.	The narrative evidence pertinent to coping is interpreted in terms of the contribution of account-making, confiding, and social support systems toward the amelioration of psychological impairment due to major losses.
**70**	Lalande (2000) Canada	To describe the impact of the July 1996 floods in Saguenay on the lives of the victims	Adults (N=30)	Interviews (life stories)	A psychologist interviews the participants. This person also made a diagnosis according to DSM-IV	Participants reported a series of problems as consequences of the floods: economic problems, interpersonal problems and feelings of loneliness, psychosocial and emotional problems, and difficulties at the workplace, physical problems, and problems related with their previous religious beliefs and adjustment difficulties. According to the interview made by the psychologist, 12 out of the 16 participants meet criteria for a current PTSD disorders two years after the flood.
**71**	Maltais (2001) Canada	to explore the impact on the health and adaptation behaviours of victims of the July 1996 floods in Saguenay	Older Adults (N=31)	Interviews	Subjective experiences on the experiences, and how it affects wellbeing	Losing home has a deep impact on the life of persons, as it is a disruption in their life. This impacts mental health. They also suggest some recommendations such as the importance to follow-up the mental health of affected people, as well as to have emergency plans. They also suggested the need of providing good information to affected people.
**72**	Maltais (2000) Canada	To describe the experiences of those affected by the July 1996 floods in Saguenay with regard the meaning of home and the problems of adjustment after the flood	Adults (N=69)	Interviews	Psychological subjective experience	People expressed the psychological impact of losing their homes, related with the loss of memories, They also commented on the problems with the insurance and the difficulties associated to adjustment.
**73**	Tapsell (2001) UK	To explore the intangible impacts and the experiences of two English communities affected by the 1998 Easter floods.	Adults (N=41)	Focus Groups	Psychological subjective experience	Many of the psychological effects attributed to the flooding are present after one year. Much of this stress is associated with the concern about future flooding's, disruption to people's life, loss of confidence in the authorities, and sense of isolation
**74**	Tapsell (2008) UK	To explore the intangible impacts and the experiences of two English communities affected by the 1998 Easter floods, four years after the flood	Adults (N=32)	Focus Groups	Psychological subjective experience	Four years later they still experienced psychological distress, highlighting the need of long term support.

Case Studies

Ref	1st_Author, Year and Country	aim	Age category	Mental Dimensions	Main Conclusions
**82**	Acharya (2006); Nepal	To describe how Nepal deal with the impact of natural disasters (special focus on floods) on Mental Health	General	Responses to ameliorate MH	This paper concludes that, although there is a Health Sector Emergency and Disaster response Plan, mental health is not adequate addressed
**84**	Choudhury (2006) Bangladesh	To highlight the activities and observation of some NGOs and some dedicated researchers in the field of psychosocial consequences of disaster preparedness in Bangladesh, during the last two decades.	General	Responses to ameliorate MH	This paper highlights the importance to include MH care in developing countries (need for training)
**76**	Cretikos (2007) Australia	To review the impact of the 2007 flooding following a severe storm from a Public Health point of view	General	Responses to ameliorate MH	It was important to include mental health in the public health surveillance system
**83**	Deshmukh (2008) India	To explain the help provided by volunteers of NGOs to people affected by July 2005 floods	General	Responses to ameliorate MH	They highlight the importance to include psychosocial needs in the event of a flooding also in developing countries. They made 5 different activities: 1) orientation of volunteers and research instruments; 2) Assessment of MH door by door; 3) Family visits (counselling) and psychiatric interventions; 4) Indirect MH interventions through community activities, and 5) Mental Health orientation to medical personnel (family physician)
**78**	Dorji (2006) Bhutan	To explain the mental health and psychosocial aspects of disaster preparedness (with a focus of earthquakes and flash floods) in Bhutan	General	Responses to ameliorate MH	They have included 4 strategies to deal with mental health and disasters: 1) inclusion of mental health specialists in the National Disasters Risk Management Committee; 2) District mental health and psychosocial counselling team; 3) Community mental health and psychosocial counselling; 4) Community volunteers and psychosocial support. All these professionals have received special training to give psychological first aid and other support.
**85**	North (2000) USA	To present the CREST program developed after the 1993 floods	General	Responses to ameliorate MH	As disaster victims seek support from trusted members of their own communities rather than mental health professionals, it is important to train these community resource persons, so they can expand the availability of mental health services. The authors presented the educational curriculum, initially designed for training mental health professionals, which was successfully adapted to train community leaders.
**80**	Otero (2006) Venezuela	To describe mental health initiatives following the December 1999 flooding	General	Responses to ameliorate MH	They developed a community mental health centre to deal with the PTSD and other comorbidities (depression and anxiety) over a 1 year period. Among 180 patients who took part in the pilot study, 74% of patients presented symptoms of PTSD. Only 53% accepted treatment. Then, they realized stigma issues related to MH disorders, a started an anti-stigma campaign at the community (community based initiatives) They also included social workers in the team.
**77**	Raguenaud (2012) France	To present the epidemiological surveillance linked to an Outreach Psychological Support Program after the 2010 flood.	General	Responses to ameliorate MH	Health providers working in affected towns were asked to complete an individual record sheet for each person who displayed psychological problems related to the storm (it was part of a free of charge outreach Psychological Support Program). The surveillance period was from 6 weeks to 6 months after the storm. A total of 227 new cases were registered. Most of them were female and one fifth had a history of psychiatric illness. Peaks on the new cases coincided with strife events: relocation of flood victims and the first visit of technical experts to assess the value of houses.

Reviews

REF	1st_Author and year	aim	Age category	Main Results
**88**	Ahern, 2005	To summarize and critically appraise evidence of published studies, covering flood events in all regions of the world, and to identify knowledge gaps relevant to the reduction of public health impacts.	General	The main result after reviewing 26 papers on floods and mental health were that the mental health impacts of flooding, especially the long-term impacts, and their principal causes, have been inadequately researched even in high-income settings;
**1**	Alderman, 2012	To review recent epidemiological evidence on the impacts of floods on human health	General	A total of 11 papers on psychosocial health were included The most common mental disorders is PTSD, followed by depression and anxiety. Psychosocial distress has a long term effect and impacts the QoL of survivors. Several risk factors were identified: degree of exposure, previous flood experience and disaster preparedness, female gender and older age, socioeconomic status, family structure-religion, social support, self-reported physical health, personality factors and direct trauma. More research is needed to clearly understand the long term effects ('anniversary reaction').
**81**	Bhamani, 2012	To review the epidemiological evidence and the implications of the rate and prevalence of mental disorder in the flood affected regions of Pakistan	General	This review concludes that the incidence of psychiatric symptoms in the aftermath of a flood is dependent on age, genders, and help received during the floods. Based on that, they suggest the need of addressing mental health disorders, especially in vulnerable population (women, children and elderly) and to ensure the availability of psychological first aid by trained professionals, awareness campaigns, and training of lay volunteers
**87**	Cherniak, 2008	To review the impact of natural disasters on the elderly (special section on mental health)	Older Adults	A total of 11 papers addressed the impact of floods on the health of the older people. Among them, just 4 dealt with mental health. There is conflicting evidence. Some papers pointed out that older people are more vulnerable to the floods, while other pointed out that they are more resilient.
**89**	de Freitas, 2012	To review the impact of floods in Public Health	General	Among the 70 papers reviewed, in 18 there was information on the impact of floods in Mental health. Results from the review suggest that the impacts of flood on mental health are related to PTSD, adjustment disorders, depression, anxiety, emotional problems, suicide, drug abuse, psychotropic use, and antisocial behaviour. They also commented on the importance of special mental health services to avoid cornification.
**90**	Du, 2010	To identify the health impacts of disasters and the underlying causes of health impacts associated with floods.	General	Among the 197 papers reviewed, they included 12 dealing with mental health consequences. Results from the review suggest that people who had experiences floods have higher risk of psychological distress than those non exposed, and a higher suicide rate. MH problems may derive from physical problems or personal losses, social disruption, and economic hardship. They also commented that mental health has not been properly addressed in the field of disaster preparedness or service delivery.
**79**	Hajat, 2005	To review the human health consequences of flooding in Europe	General	A total of 17 papers on mental health were reviewed. Flooding is associated with increased rates of the most common mental disorders. Aside from the experience of being flooded, many mental health problems stem from the troubles brought about displacement, damage to the home or loss of familiar possessions, and the stress involved in dealing with builders, insurance during the aftermath. Lack of insurance (or under-insurance) was a common factor exacerbating the impacts of floods. The review also pointed out the need for mental health services as a strategy to reduce the risk
**86**	Kolves, 2013	To systematically analyse the existing literature on the potential impact of natural disasters (including floods) on suicidal behaviours	General	Only 3 papers were found. The evidence suggests an increase in suicidal ideation and attempts during the aftermath. However, it also seems that greater amounts of time spent volunteering in flood recovery efforts were associated with increased feelings of belonging, decreasing the desire of suicide.
**91**	Kovats, 2004	To review the impact of climate change, with a special focus on floods and heatwaves , on health	General	A total of 5 papers focused on mental health were included. This review concluded that Mental health effects directly occurring due to the experience of being flooded, or indirectly during the restoration process. Regarding public health responses the authors highlighted the importance of post-flood counselling. Visits by health workers or social workers to vulnerable people (elderly, disabled, etc.)
**8**	Lowe, 2013	To review the risk factors for morbidity and mortality effects pre-, during and post-flood may aid the appropriate targeting of flood-related adverse health prevention strategies.	General	A total of 45 studies were including. Fourteen had as a dependent variable psychological health. During floods, females, elderly and children appear to be at greater risk of psychological and physical health effects. Post-flood, females appear at greater risk of psychological health effects. Other risk factors include: previous flood experiences, greater flood depth or flood trauma, existing illnesses, low education or socio-economic status and social connectedness
**5**	Stanke, 2012	To review the epidemiological evidence about the mental health impacts on people who have been affected by flooding and to identify current gaps	General	They select 48 papers out of 3585 who met the inclusion criteria. Their review concluded that flooding affects people of all ages, and that can exacerbate or provoke mental health problems. The authors also highlighted the importance of secondary stressors.
